# Primary pulmonary marginal zone lymphoma: an unusual cause of pulmonary infiltrates

**DOI:** 10.1002/rcr2.806

**Published:** 2021-06-29

**Authors:** Robert Smyth, John Mark Sloan, Eric Burks, Finn Hawkins

**Affiliations:** ^1^ Department of Pulmonary and Critical Care Medicine Boston University School of Medicine, Boston Medical Center Boston MA USA; ^2^ Department of Hematology and Oncology Boston University School of Medicine, Boston Medical Center Boston MA USA; ^3^ Department of Pathology and Laboratory Medicine Boston University School of Medicine, Boston Medical Center Boston MA USA

**Keywords:** Extra‐nodal lymphoma, MALToma, marginal zone, pulmonary lymphoma, rare lung tumour

## Abstract

Primary pulmonary extra‐nodal marginal zone lymphoma of mucosa‐associated lymphoid tissue (MALT lymphoma), also known as bronchus‐associated lymphoid tissue (BALT lymphoma), is the most common primary pulmonary lymphoma but is rare (<1%) among all non‐Hodgkin lymphomas and among pulmonary neoplasms in general. We herein report the case of a 59‐year‐old male who presented with stable exertional dyspnoea and persistent lung infiltrates who was referred to our hospital for further assessment. A computed tomography (CT)‐guided core biopsy was performed showing a dense lymphoid infiltrate, with further testing revealing the diagnosis of pulmonary MALT lymphoma. This uncommon lung tumour is usually seen in older adults and typically associated with a relatively indolent course. Rituximab, an anti‐CD20 antibody, has been shown to be effective in up to 70% of cases.

## Graphical abstract

A middle‐aged male presents with dyspnoea and multifocal consolidations. Biopsy shows pulmonary MALT lymphoma, a rare lung tumour.

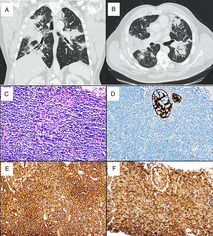

## Introduction

Primary pulmonary mucosa‐associated lymphoid tissue (MALT) lymphomas of the lung account for the majority of primary pulmonary lymphomas, but account for less than 0.5% of all primary lung neoplasms and a similarly small proportion of all lymphomas. They are relatively indolent tumours and their appearance is radiographically similar to far more common lung diseases. Therefore, a high index of suspicion is needed with biopsy and appropriate staining to confirm the diagnosis. Here, we report a case of primary pulmonary MALT lymphoma in a middle‐aged male with persistent pulmonary infiltrates.

## Case Report

A 59‐year‐old male presented for evaluation of chronic exertional dyspnoea. He denied cough, sputum, or other infectious symptoms, and no prior travel. His weight was unchanged. He was previously incarcerated and had smoked illicit substances including cocaine as well as a 15 pack‐year smoking history. There were no known environmental exposures or tuberculosis (TB) contacts. On examination, he appeared well, oxygen saturation in room air was reduced at 93%, his chest was clear to auscultation, and palpable lymphadenopathy or organomegaly was absent.

**Figure 1:**
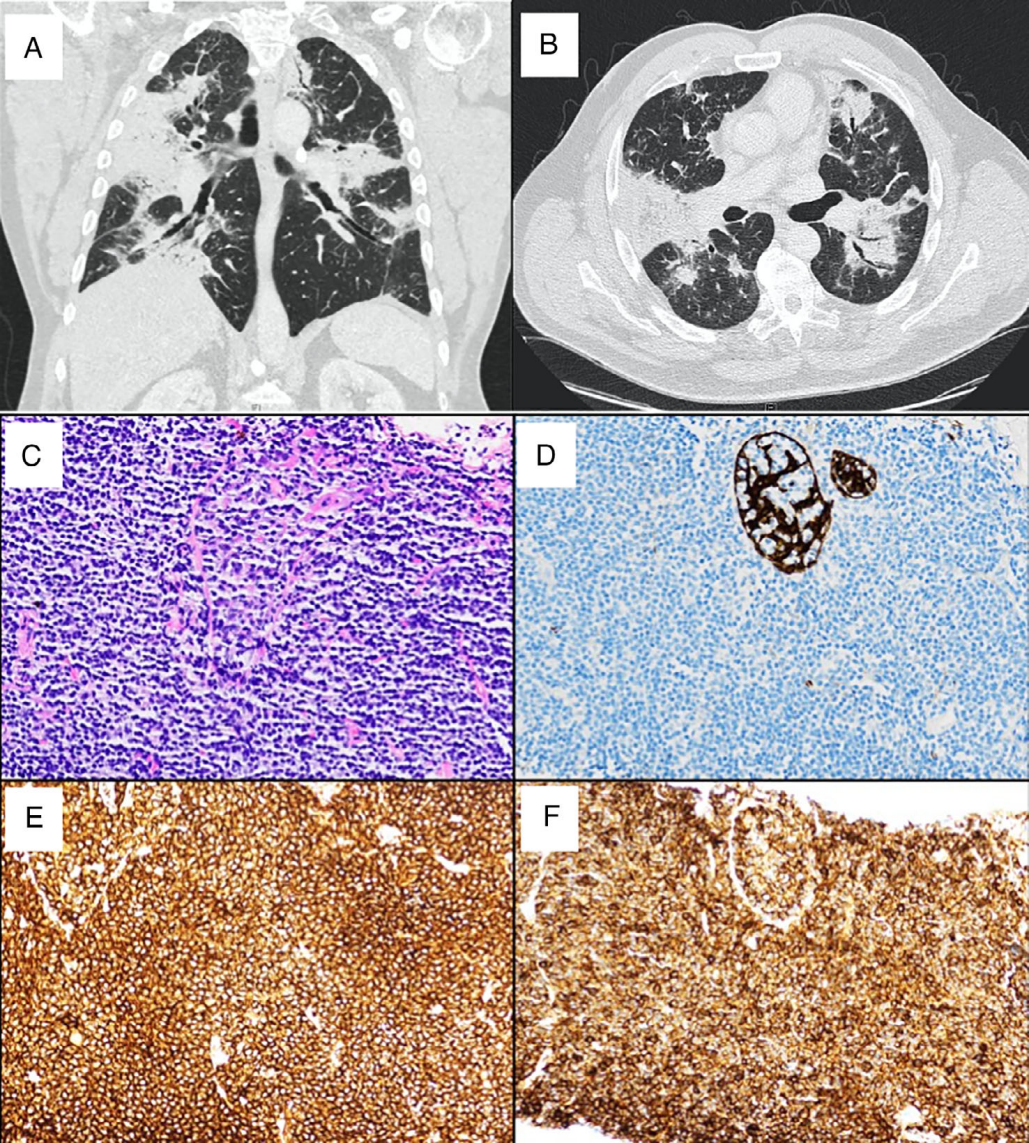
(A, B) Coronal and axial sections of non‐contrast chest computed tomography (CT) scan demonstrated dense, multifocal consolidations around the airways. Incidental note of calcified mediastinal node from prior infection. (C) Haematoxylin and eosin (H&E) stain of CT‐guided lung biopsy identified a dense lymphoid infiltrate. (D–F) Immunolabelling of lung tissue with antibodies against cytokeratin stain AE1/AE3 (D) showing a lymphoepithelial lesion, CD20 (E), and CD43 (F)

Routine laboratory tests were normal, with negative connective tissue serologies, HIV, and QuantiFERON®‐TB GOLD PLUS (Qiagen, USA). A serum protein electrophoresis identified a suspicious band in the IgM lane on immunofixation. A CT of the chest, abdomen, and pelvis demonstrated multifocal dense consolidations throughout all lobes of the lung (Fig. 1A, B) without mediastinal or extra‐thoracic lymphadenopathy. Pulmonary function testing showed moderate restriction and reduction in diffusing capacity.

A bronchoscopy demonstrated normal bronchial mucosa and sterile bronchial washings. Flow cytometry performed on the bronchoalveolar lavage fluid identified kappa‐restricted B cells with co‐expression of CD43. A CT‐guided core biopsy of the left lower lobe (Fig. 1C) showed a dense lymphoid infiltrate comprised of small cells without plasmacytic differentiation. Immunohistochemistry for cytokeratin AE1/AE3 (Fig. 1D) highlighted lymphoepithelial lesions while additional immune cell markers showed the neoplastic lymphocytes to stain with CD20 (Fig. 1E) with few admixed reactive CD3‐positive lymphocytes and aberrant co‐expression of CD43 (Fig. 1F). Flow cytometry additionally showed the B cells were kappa light chain restricted. A diagnosis of primary pulmonary MALT lymphoma was made and the patient was commenced on rituximab therapy with improvement in symptoms.

## Discussion

The lung incorporates many lymphatics including MALT specific to the lung or bronchus (bronchus‐associated lymphoid tissue, BALT). BALT is comprised of organized lymphoid tissue found in the mucosal region of the distal airways and composed primarily of B cells [1]. It is considered important in immune response whereupon exposure to inhaled antigens causes several morphological and functional changes within proliferating B cells, a visible marginal zone with stimulation of various cytokines and IgA [2]. It is postulated that chronic antigen stimulation may lead to clonal B‐cell expansion and malignant transformation.

Primary pulmonary marginal zone lymphoma (PP‐MZL) or MALT lymphoma is a rare extra‐nodal lymphoma within the primary pulmonary B‐cell lymphoma group of diseases. It usually affects older adults (median age 66 years) [3] and is associated with autoimmune disease in up to one‐third of cases [4]. Serum findings include monoclonal gammopathy with a serum monoclonal IgM spike in up to 30% of cases. Chest CT usually shows unilateral disease with solitary or multiple large nodules, although consolidation and air bronchograms have been described and should raise suspicion in the appropriate clinical context. Most lesions demonstrate homogenous increased fluorodeoxyglucose (FDG) avidity on positron emission tomography (PET)‐CT. Mediastinal adenopathy is unusual. Diagnosis is usually possible with transbronchial biopsy with a reported sensitivity of 88% [5]. Transbronchial cryobiopsy may be a potential alternative to surgical lung biopsy in those whose initial sampling is indeterminate. Histologically, it appears as a morphologically heterogeneous infiltrate of dense lymphoid tissue often disrupting the lung architecture with infiltration of the bronchial mucosal epithelium forming foci of lymphoepithelial lesions [6]. Detection of light chain restriction is also supportive. Due to its indolent nature, the proliferation index Ki‐67 is typically low (<10%).

This diagnosis represents a particular challenge to the pulmonologist. The imaging findings are non‐specific and are more often seen in other more common diseases such as organizing pneumonia, eosinophilic pneumonia, and multifocal adenocarcinoma. Furthermore, a number of closely related benign and malignant diseases can be mistaken for PP‐MZL. Nodular or diffuse lymphoid hyperplasia, lymphocytic interstitial pneumonia, mantle cell lymphoma, follicular lymphoma, or pulmonary spread from more common forms of MALT lymphoma all need to be considered. Median survival is generally over 10 years with rituximab used to stabilize or reduced disease burden and relapse is common.

In summary, we present a challenging case of extensive pulmonary infiltrates and PP‐MZL. Differential diagnosis in these cases is broad with many clinical and radiologic overlap, therefore high‐quality tissue samples are needed for definitive diagnosis.

### Disclosure Statement

Appropriate written informed consent was obtained for publication of this case report and accompanying images.

### Author Contribution Statement

Robert Smyth wrote the manuscript. John Mark Sloan, Eric Burks, and Finn Hawkins were involved in the interpretation of the data. John Mark Sloan, Eric Burks, and Finn Hawkins provided expertise and feedback. All authors drafted the article, revised it critically for important intellectual content, and approved the final version to be submitted.
